# Monolithic integration of a 10 μm cut-off wavelength InAs/GaSb type-II superlattice diode on GaAs platform

**DOI:** 10.1038/s41598-022-15538-3

**Published:** 2022-07-08

**Authors:** D. C. M. Kwan, M. Kesaria, J. J. Jiménez, V. Srivastava, M. Delmas, B. L. Liang, F. M. Morales, D. L. Huffaker

**Affiliations:** 1 School of Physics and Astronomy, Cardiff University, The Parade, Cardiff, CF24 3AA UK; 2 Department of Materials Science and Metallurgical Engineering and Inorganic Chemistry, Faculty of Sciences, University of Cádiz, 11510 Puerto Real, Cádiz, Spain; 3 IMEYMAT: Institute of Research On Electron Microscopy and Materials, University of Cádiz, 11510 Puerto Real, Cádiz, Spain; 4 California NanoSystems Institute, University of California, Los Angeles, CA 90095 USA; 5 Present Address: IRnova AB, Electrum, 236 - C5, 164 40 Kista, Sweden; 6 Present Address: Electrical Engineering Department, The University of Texas at Arlington, Arlington, 76019 USA

**Keywords:** Materials science, Nanoscale materials, Two-dimensional materials

## Abstract

At room temperature, a 10 µm cut-off wavelength coincides with an infrared spectral window and the peak emission of blackbody objects. We report a 10 µm cut-off wavelength InAs/GaSb T2SL p-i-n diode on a GaAs substrate with an intentional interfacial misfit (IMF) array between the GaSb buffer layer and GaAs substrate. Transmission electron microscopy and energy-dispersive X-ray spectroscopy revealed that the heterostructure on GaSb-on-GaAs is epitaxial, single-crystalline but with a reduced material homogeneity, extended lattice defects and atomic segregation/intermixing in comparison to that on the GaSb substrate. Strain-induced degradation of the material quality is observed by temperature-dependent current–voltage measurements. The T2SL with the IMF array appears as a potentially effective route to mitigate the impact of the lattice mismatch once its fabrication is fully optimized for these systems, but additional strain compensating measures can enable a low cost, scalable manufacturing of focal plane arrays (FPA) for thermal imaging cameras for spectroscopy, dynamic scene projection, thermometry, and remote gas sensing.

## Introduction

The past decade has seen a deepened interest in infrared (IR) technologies which have been galvanized by advances in the growth and fabrication of emerging IR materials. Notable developments have been made in III-V and II-VI compound semiconductors^1,2^, perovskites^3,4^, 2D-materials^5,6^, metamaterials^7,8^, and colloidal quantum dots^9,10^.

The II-VI compound semiconductors, HgCdTe (MCT) or variants thereof, are the most mature of the aforementioned material systems. Long minority carrier lifetimes, high absorption coefficients, and good bandgap tuneability have made MCT an attractive material system for IR devices^11^. However, the high costs, poor uniformity, and limited theoretical performance due to Auger recombination and the low electron effective mass associated with these devices have been seen to limit their industrial potential^12^. III-V compound semiconductor devices represent a lower cost, high-performance alternative but costs remain high compared to less mature technologies such as colloidal quantum dots. By replacing the commonly used but expensive GaSb or InAs substrates with cheaper GaAs alternatives, it is possible to substantially reduce these costs and access a range of performance-enhancing advantages as discussed below^13–15^. However, due to the considerable lattice mismatch of 7.8% between GaSb and GaAs, the critical thickness of such layers is predicted to be only a few monolayers (ML) before the generation of threading dislocations^16^_,_ which have been shown to have a detrimental effect on device performance^17^. Fortunately, the degradation of the material quality due to the non-native GaAs substrate can be mitigated by the interfacial misfit (IMF) array, developed by Huang et al.^18,19^. The IMF consists of a periodic array of 90° dislocations at the GaSb-on-GaAs interface (IF) which prevent the propagation of threading dislocations into the device's active region. This technique has been proven to reduce the density of threading dislocations from 10^8^ to 10^9^ cm^-2^ down to 10^5^ cm^-2^^20^. Furthermore, the IMF array can also be used to compensate for the internal strain within heterostructures such as devices incorporating distributed Bragg reflectors^21,22^. The IMF array has thereby enabled the growth and cost reduction of a range of IR devices^23–26^.

The type-II superlattice (T2SL), a periodic stack of two or more semiconducting layers theorized by Esaki et al.^27^, is considered one of the most promising III-V based alternatives to MCT due to suppressed Auger recombination, reduced tunneling currents, and impressive advances in bandgap engineering^2,12^. Furthermore, the bandgap tuneability of the T2SL from around 3.5–30 μm, achieved by tuning the thicknesses of the constituent layers, has enabled the development of a variety of devices including photodetectors^28–30^, LEDs^31,32^, lasers^33,34^, and phototransistors^35,36^. The IMF array has already proven to be a beneficial innovation for T2SL IR devices, bringing notable advantages such as larger wafers, semi-insulating properties^15^, increased quantum efficiency^13,14^, and the opportunity for obtaining optically immersed detectors^37–41^, in addition to reduced costs.

IR radiation around 10 μm in wavelength is of particular interest for many applications in imaging, spectroscopy, dynamic scene projection, thermometry, and gas sensing. This is largely due to its coincidence with both the long-wavelength infrared (LWIR) spectral window and the peak emission of blackbody objects at room temperature. Furthermore, LWIR detectors, lasers, and LEDs are required for applications such as ethanol^42^ and freon^43^ sensing. However, there are also considerable challenges associated with this wavelength due to the small (~ 0.12 eV) bandgap leading to high thermal currents. It is thought that T2SLs on GaAs, grown using the IMF array, can provide a low cost, high-performance solution to the demands of devices in the LWIR regime. These expectations have been buoyed by recent reports in which LWIR T2SL-based photodetectors on GaAs are comparable to or even outperform MCT devices in terms of detectivity at temperatures around 200 K^37,38,41^.

Despite initial successes, detailed characterization of the IMF array itself, and its impact on SL structure and device performance, has so far been limited in LWIR T2SLs^15,37,41,44–46^. LWIR InAs/GaSb T2SL detectors have been demonstrated on GaAs substrates in both photovoltaic^13^ and photoconductive^39^ operation but these reports made use of thick, and therefore expensive, buffer layers (> 4 μm) rather than the IMF array. Furthermore, electrical measurements of LWIR T2SLs on GaAs that make use of the IMF array have, so far, been limited to resistivity and carrier concentration measurements and do not include dark current characterization^15,46^. Further studies are therefore required to understand the effect of the IMF array on the material quality and device performance of LWIR InAs/GaSb T2SLs on GaAs substrates.

This report aims to determine the efficacy of the IMF array by growing a p-i-n diode with a 12 ML InAs/ 4 ML GaSb (12/4) SL on a GaAs substrate. We have demonstrated a lower diffusion current in a 12/4 SL at 150 K in comparison to the most commonly used 14/7 SL^47^. Enhancement in electron–hole wavefunction overlap and absorption coefficient in the 12/4 SL suggests its suitability for high performance 10 μm optoelectronic applications such as photodetectors, LEDs, and lasers^47^. More recently, we have also reported on the structural and optical quality of a 12/4 SL grown directly on GaAs substrates^44^. Here, we advance this previous report through growth, fabrication, and comparative characterization of 12/4 SL p-i-n diodes on GaAs and GaSb substrates. Transmission electron microscopy (TEM) measurements are carried out to observe the resulting heterostructures directly and thus study relevant features in each sample such as the potential existence of lattice defects, the crystallinity of the T2SLs, or their chemical homogeneity. For this last feature, we employed energy-dispersive X-ray spectroscopy (EDX) under scanning-TEM (STEM) conditions. Temperature-dependent current–voltage measurements were performed to compare device performance and identify the limiting dark current mechanisms in each sample. Ultimately, the results can be used to characterize the impact of the IMF array for optimization of future device designs.

## Results

### Optical and surface characterization

**Figure 1:**
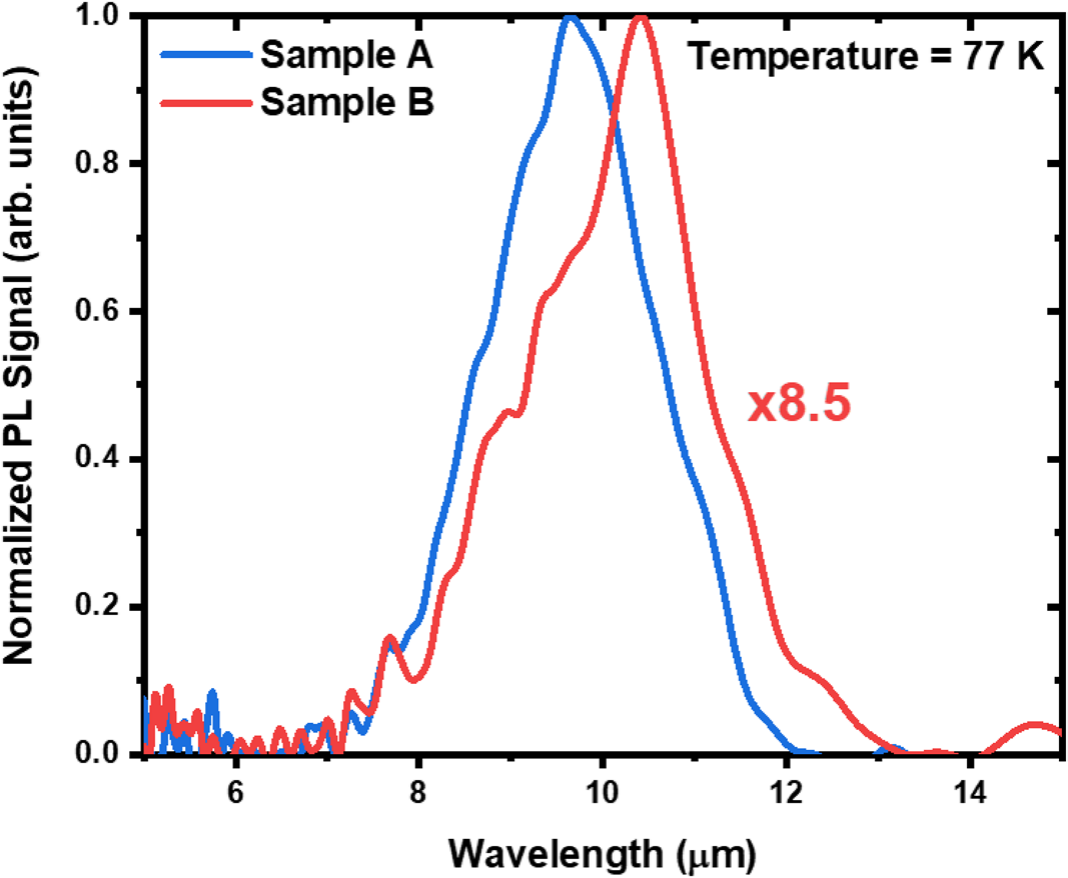
Normalized PL comparison at 77 K for Samples A and B.

Two 12/4 SL reference samples were grown by Molecular Beam Epitaxy (MBE) on a GaSb substrate (Sample A) and a GaAs substrate using the IMF array (Sample B). The structures of these samples have been reported previously^45,47^. Photoluminescence (PL) measurements, shown in Fig. 1, were performed on Samples A and B at a temperature of 77 K. Figure 1 shows that both samples register clear PL profiles at 77 K but the max PL intensity of Sample A is around 8.5 times that of Sample B. As reported in our previous paper, the degradation of the PL signal measured from Sample B is likely a result of inferior material quality resulting from the lattice mismatch of the non-native substrate. As Table 1 shows, the full width at half maximum (FWHM) of the PL Profile of Sample B is broader than that of Sample A. While the noise associated with the PL profiles is likely to be too large for quantitative analysis, the broadening in the case of Sample B is likely related to material phenomena such as fluctuations in well-width, alloy compositions, and interface roughness^48^. These material phenomena are likely the cause of the greater route mean square (RMS) surface roughness over a 4 μm × 4 μm area in Sample B (Table 1), as measured by atomic force microscopy (AFM). This further corroborates previously reported HR-XRD measurements^45^ which show Sample B has an XRD FWHM four times larger than Sample A, indicative of greater material inhomogeneity.

**Table 1:** Parameters extracted from PL and AFM measurements.

Sample	Substrate	λ_max_ (μm)	PL FWHM (meV)	RMS roughness (nm)
Sample A	GaSb	9.85	24.0	3.2
Sample B	GaAs	10.35	29.2	223.2

### Transmission electron microscopy

In our previous study of Samples A and B, some key differences were found in the material characterizations of the resulting InAs/GaSb T2SLs depending on the substrate selected (i.e. GaSb or GaAs). Among the reasons given to possibly explain such differences was that the sample on GaAs could contain a higher density of defects than the sample on native GaSb. Hence, this might contribute to differences in their PL response, since defects such as vacancies or threading dislocations can function as non-radiative recombination centers, as has been observed in other materials^49–51^. Transmission electron microscopy is a very powerful tool to ascertain such hypotheses, and thus, we resorted to this technique to observe directly the heterostructures in cross-section preparations of these samples. Under a proper orientation of the sample with respect to the electron beam, and using a suitable configuration in the microscope, a lot of information about materials, such as their crystal quality, can be extracted.

**Figure 2:**
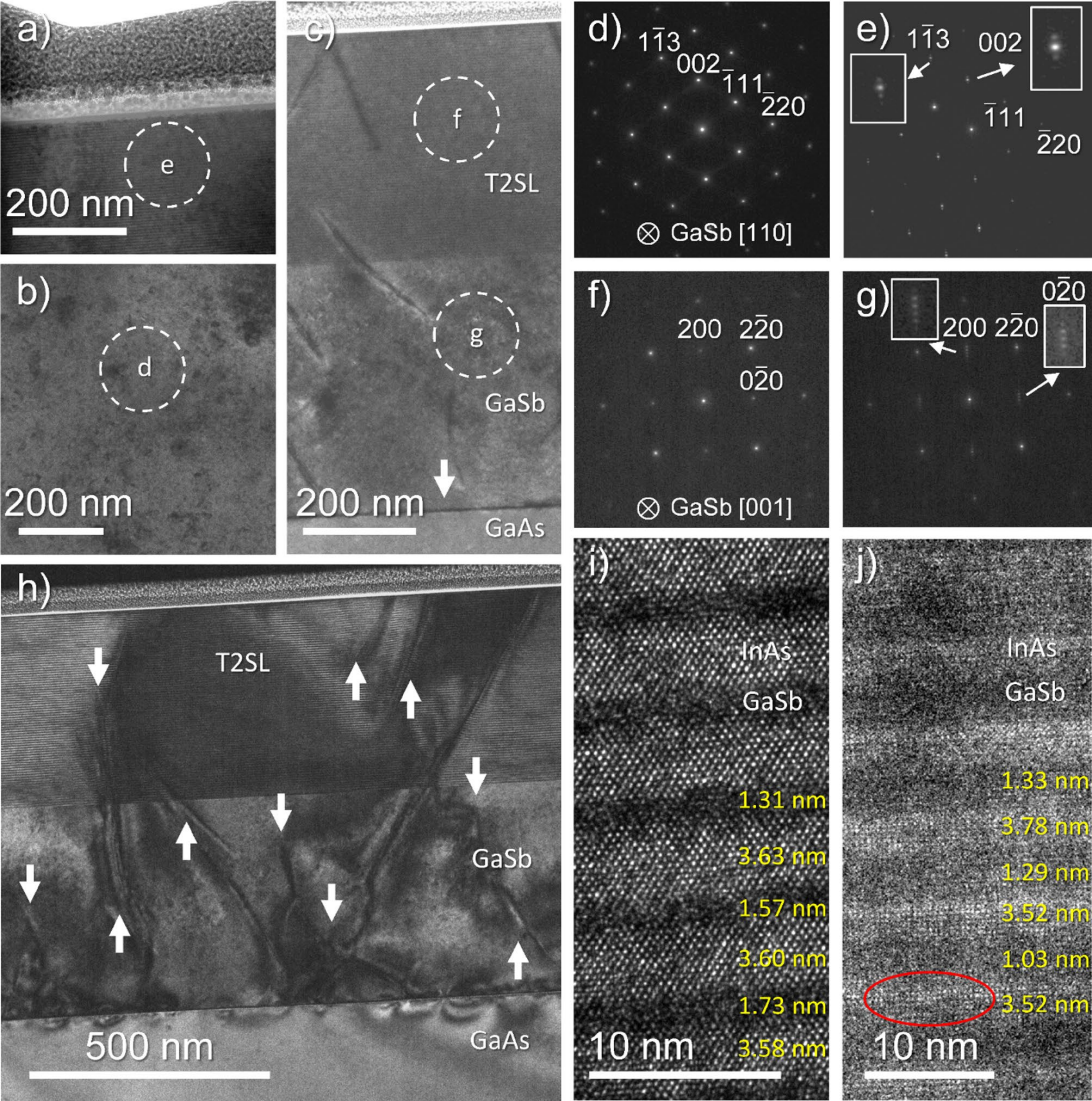
CTEM micrographs from a cross-section of sample A: top layers of the T2SL (**a**) and GaSb substrate (**b**). CTEM image of the whole heterostructure in sample B observed in cross-section (**c**). White circles mark the approximated regions of included SAED patterns (**d**)-(**g**). 2B-DC-BFTEM micrograph of sample B (**h**) and HRTEM images from the middle T2SL region in samples A (**i**) and B (**j**), including some thickness measurements. In the latter, a red circle is included as a guide to the eye to identify some atomic columns more clearly.

Figure 2 compiles several results about the TEM characterization of Samples A and B. Figure 2a–c are low-magnification conventional TEM (CTEM) micrographs, taken from Samples A and B after tilting the samples until the electron beam is parallel to the GaSb [110] (Sample A, a)-b)) or GaAs [001] (Sample B, c)) zone axes and inserting an objective lens aperture. The IMF array can be identified in Fig. 2c as the dark line separating GaSb and GaAs, which is indicated with an arrow. In terms of the structural quality of the obtained heterostructures, some differences can already be found between them even at this relatively low magnification. On the one hand, Sample A reveals a very homogeneous contrast throughout the T2SL and the buffer layer, which means that no extended lattice defects are present. On the other hand, in Sample B, some features, which will be analyzed shortly, can be recognized as line-shaped, dark formations starting at the GaSb-on-GaAs IF or appearing at apparently random spots of the heterostructure, some of them being straight lines that form an angle with respect to the growth plane. Representative selected-area electron diffraction patterns (SAEDs) from regions like the ones marked in these micrographs are included in Fig. 2d–g. They are useful to find some positive features common to both samples that are well-known in these systems, such as the presence of single-crystalline, epitaxially related materials (since their respective reflections are aligned and discrete and they do not contain diffraction rings or diffuse signals).

As for the GaAs substrate in Sample B, the same conclusions as those about GaSb could be extracted from observing its associated SAED pattern (not shown), since it exhibited an arrangement of diffraction spots very similar to the one present in Fig. 2f. The superlattice layers can be identified in their associated SAED patterns (Fig. 2e and g for Samples A and B respectively), since they exhibit features that are typical in these materials when they are observed under electron diffraction conditions. Namely, additional satellite reflections around the brightest spots can be found along the growth direction, allowing their identification as a long-period structure formed by the repeated combination of InAs/InSb/GaSb layers^52^. Insets magnifying some of these signals (marked with arrows) are included to observe them more easily.

The presence of lattice defects in Sample B is more evident in Fig. 2h, which is another low-magnification micrograph showing the heterostructure of this sample. However, in this case, the image was obtained under two-beam and bright-field diffraction contrast TEM conditions (2B-BF-DCTEM) to enhance the visibility of defects, some of them being marked with white arrows in this figure. This is the only image of the figure taken with the electron beam not parallel to a zone axis. In this picture, the discontinuities observed are generating distortions throughout the superlattice, and they exhibit a contrast that is comparable to that exhibited by features found in other works in the literature for the same material or other similar systems. That is, threading dislocations and stacking faults along specific glide planes^53,54^. The former seems to be coming from some random spots of the GaSb-on-GaAs interface where, in principle, only 90° misfit dislocations were intentionally formed in order to generate an IMF array able to allow the strain relaxation of GaSb without formation of threading dislocations^55^. If any threading dislocations appeared, in this context and in accordance with their nature, they should terminate mainly at the surface of the heterostructure or get annihilated by reaching other dislocations. Since these linear defects begin at the GaSb/GaAs interface, their origin must be linked to the presence of some 60° misfit dislocations at that area. In contrast to 90° dislocations, these do allow the formation of threading segments in GaSb^55^. Although 90° misfit dislocations are more efficient for strain accommodation than 60° misfit dislocations, the formation of the latter can still happen depending, for example, on parameters like the GaSb growth temperature^56^. Thus, assuming that the GaSb/GaAs pair was not prepared under fully optimized conditions for the subsequent growth of the superlattices, it is reasonable to conclude that threading dislocations which contain 60º misfit dislocations are more likely to appear at random regions of the IMF array. This would thus imply that the strain of the GaSb-on-GaAs heterostructure was not accommodated following a uniform fashion, which would consequently impact the subsequent growth of the T2SL. As a result, there would be some alterations in the ordered deposition of the elements which constitute the T2SL. Considering the results reported on a somewhat comparable superlattice system^53^, it is reasonable to assume that the previously propagating defects would induce a mechanical strain within the T2SL that would lead to atomic intermixing at some spots of the deposited superlattice, as well as to additional defects like stacking faults. This is what is observed in Sample B, as some of the features marked in Fig. 2h show. Considering works like the ones previously cited, we can assume that a fraction of the misfit dislocations formed at the IMF array of this sample is 60° dislocations, which motivated a more disordered growth fashion of the heterostructure in some regions. Thus, alternative strain relaxation methods like the formation of additional lattice defects took place throughout the whole heterostructure. Nevertheless, it is worth remarking that, since a fraction of these defects is ending at the T2SL-on-GaSb interface according to Fig. 2h, it appears that the GaSb layer is also partially working as a filter for some of the dislocations, a role that has been fulfilled in the literature by other materials for the growth of GaSb with improved quality^57^. However, those other defects that propagate towards the superlattice and can even reach the surface are possibly related to the results obtained in the characterization by Nomarski microscopy of these same samples in our previous paper. Furthermore, some local bending contours are found along the GaSb-on-GaAs interface. Due to the characteristics of the IMF array previously described, it is reasonable to assume that such contours appear as a consequence of the relaxation pathway selected for the accommodation of the lattice mismatch that exists between GaSb and GaAs, which motivates the further formation of other lattice defects and their potential propagation towards the other materials epitaxially grown on top of them, as is the current case with the deposited superlattice. Nevertheless, neither superlattice looks significantly rough when these cross-sections are observed at higher magnification and far away from any defective regions, and the transitions from one layer to another are clear at least for the main components of each superlattice (i.e. InAs and GaSb). This is depicted in Fig. 2i and j, which are high-resolution TEM (HRTEM) micrographs taken from the first bilayers of the T2SL in Samples A and B, respectively. Both InAs and GaSb can be distinguished clearly in these HRTEM micrographs. Under the current imaging conditions, the brightest layers are InAs and the darkest ones are GaSb, whereas the InSb interlayers should be present as much thinner films between both materials.

For a cleaner presentation of the figure, and since the resolution of the HRTEM images obtained for Sample B in Fig. 2j is very low to pinpoint these interlayers with confidence (their theoretical thickness, which is about 0.36 nm, is close to the 0.25 nm point resolution of the employed microscope in transmission mode and the images were not taken at the highest magnification possible), only the thickest materials (i.e. InAs and GaSb) are reliably labeled in both figures, whereas the presence of InSb will be confirmed later indirectly using another technique. Furthermore, in Fig. 2j, some atomic columns are encircled as a guide to identify them more easily in the micrograph. In this sense, since each image depicts the superlattice projected along a different zone axis, the atomic stacking sequence changes. Some measurements of individual layers are included in Fig. 2i and j to indicate the thicknesses found for InAs and GaSb. It can be observed that the resulting values are reasonably close to the theoretical ones at least for the InAs films (i.e. about 3.6 nm), whereas those obtained for GaSb look more deviated with respect to the expected thickness (i.e. about 1.2 nm). Nevertheless, it is worth remarking that pinpointing the interfaces in these images is not trivial because InSb is not being well resolved spatially, so deviations in the measurements are likely to happen in these images. Also, although the layers are not significantly rough in these images, they are still rough enough to further complicate the identification of the interfaces. However, the measurements available allow us to conclude that, although the thickness of the layers is not always equally homogeneous, the InAs films are always thicker than the GaSb ones, even though some regions in Fig. 2j appear to show the opposite situation for Sample B (i.e. GaSb thicker than InAs), which would be contrary to the situation expected in a 12/4 InAs/GaSb T2SL. The measurements shown are further supported by the compositional maps shown later, which also reveal values for the bilayer thickness that lie reasonably close to the ones expected in these heterostructures (i.e. about 5 nm)^45^. These regions with a more irregular bilayer thickness found by HRTEM, together with the compositional maps, can explain the slight disparity in the period thickness which was reported in that previous work. Nevertheless, this does not change the general conclusion that can be reached when Fig. 2 is considered as a whole. That is, using a GaSb buffer grown with an IMF array alone is not a suitable enough solution to obtain an InAs/GaSb T2SL on a GaAs substrate without the formation of a significant amount of lattice defects within its microstructure. Consequently, this results in the growth of a superlattice with worse optical and material qualities than comparable GaSb-based alternatives, as proved in the previous analysis of these samples^46^.

**Figure 3:**
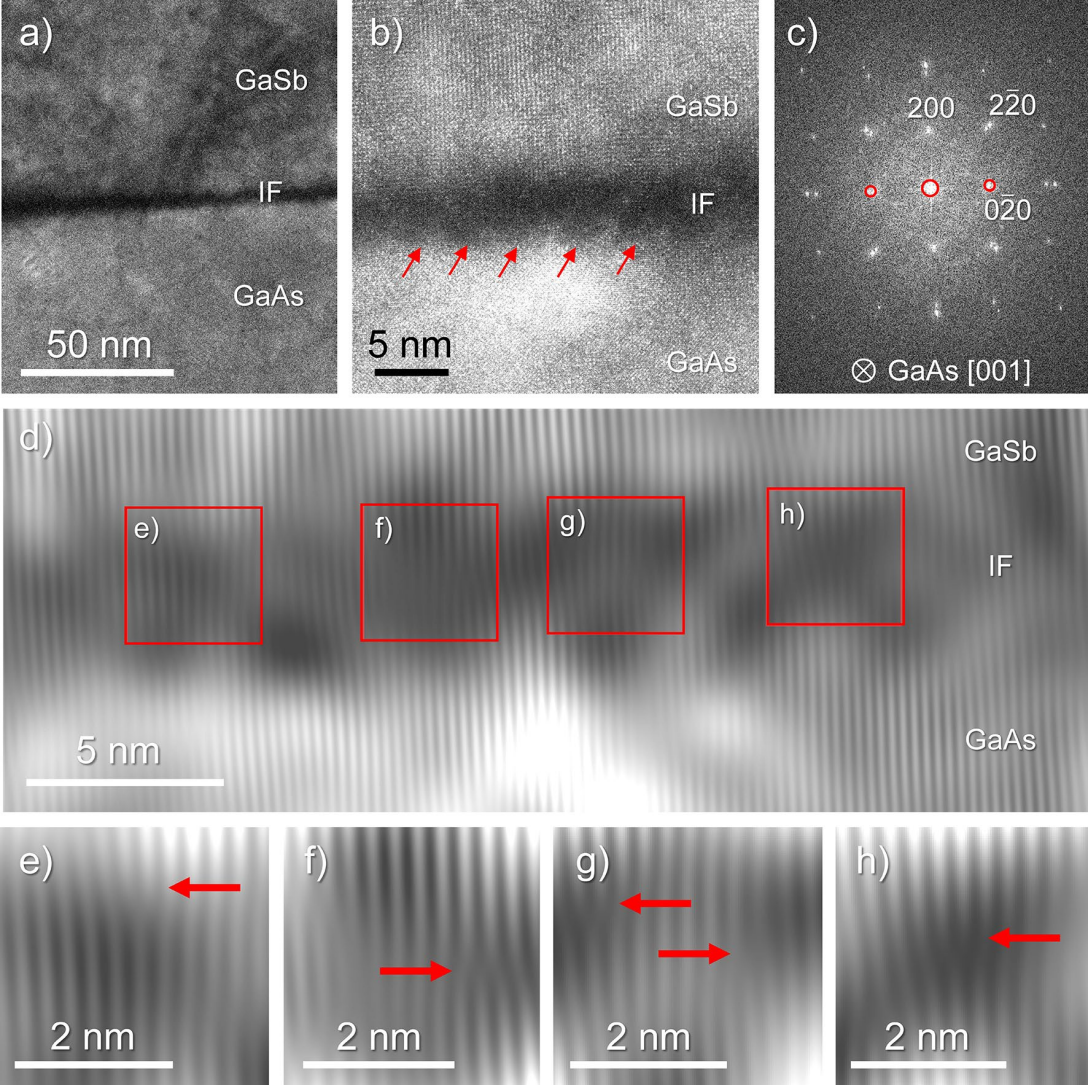
From the GaSb-on-GaAs interface in Sample B: Low magnification CTEM micrograph (**a**), HRTEM image (**b**) and associated reduced FFT of the latter (**c**). Crop of resulting image after filtering the spots indicated in the original FFT and calculating its inverse FFT (**d**) indicating the areas zoomed for better identification of half-planes (**e**)-(**h**).

The formation of IMF arrays is one possible approach in the design of these heterostructures to grow T2SL systems with improved quality. As commented previously, the IMF array in Sample B requires additional adjustments in future iterations to enhance the quality of the superlattices deposited on the GaSb buffer. Nevertheless, with Fig. 3, we can demonstrate that the IMF array in this sample was successfully formed with reasonably good quality since it contains misfit dislocations distributed in an approximately periodic fashion throughout the GaSb-on-GaAs interface. Figure 3a is a low magnification CTEM micrograph centered around the GaSb-on-GaAs interface. Here, a dark line (the interface, labelled as IF) is found separating both materials. By observing the interface at a higher magnification, as depicted in the HRTEM micrograph in Fig. 3b, atomic columns from both materials, as well as an array of rough circle-shaped, dark features distributed along with the IF (and thus perpendicular to the growth direction), some of which are marked with arrows, can be distinguished. Both pictures were taken with the electron beam parallel to the same zone axis as Fig. 2c, f, g, and j. This is proved when the reduced fast Fourier transform (FFT) of the region shown in Fig. 3b is calculated. The resulting arrangement of signals, presented in Fig. 3c, is comparable to the SAED pattern in Fig. 2f, but now the contributions from both GaAs and GaSb can be identified as square-shaped arrays of spots. The largest one belongs to GaAs and the smallest one to GaSb since the lattice constant of the former is lower than that of the latter and the FFT shows signals from these materials in the reciprocal space. The arrays contain only isolated reflections from each material that are aligned to each other, which proves the same heteroepitaxial relation as at the T2SL-on-GaSb interface.

Regarding the IMF array, its presence is confirmed by the circular features previously marked in Fig. 3b. The contrast of these formations is reasonably comparable to those observed in other works^16^. Nevertheless, since the selected zone axis might not be the most optimum choice to achieve a good contrast of this IMF array in Sample B, we resorted to an additional tool to facilitate its observation. Namely, we filtered the FFT by carefully selecting only the central spot as well as the signals from the {020} planes, which are perpendicular to the IF and are marked with red circles in Fig. 3c. Once this selection is carried out, the inverse FFT is calculated, and thus a new image formed by contributions from only the aforementioned spots is obtained. Figure 3d is a crop of the inverse FFT that is obtained after filtering Fig. 3c as explained, subsequently centered and zoomed around the GaSb-on-GaAs interface to get a better overview of this region. Several areas of this filtered image, which are roughly aligned along with the IF, are approximately marked with squares and zoomed-in Figs. 3e–h. In these areas, similar features in the GaSb-on-GaAs IF can be observed and are marked with arrows. These arrows are pointing to half-planes^58^ that are parallel to the growth direction and are mostly linearly distributed along the middle of the IF. Thus, we can assume that this region would correspond to a sharp transition from the GaAs substrate to the beginning of the GaSb buffer layer. These half-planes can be associated with pure edge dislocations, thus revealing the presence of the misfit dislocations that constitute the IMF array along with the GaSb-on-GaAs interface. Although a statistical study of the distance between these dislocations is not carried out in this work, it is reasonable to assume, considering Fig. 3b and d together with the scales provided, that the average periodicity of these features must lie between 2 and 6 nm. In any case, it is possible to conclude that an IMF array has been grown in Sample B as intended, even though it is not fully preventing the generation of additional lattice defects in the GaSb buffer that are even able to propagate towards the superlattice.

**Figure 4:**
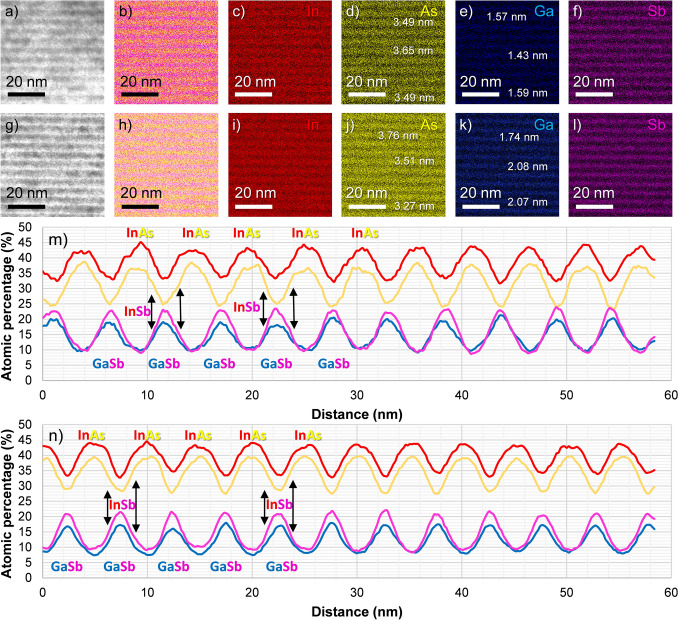
For Samples A (**a**)-(**f**) and B (**g**)-(**l**) and from left to right: STEM-HAADF image from a region around the middle layers of the T2SL, spectrum image combining atomic percentage maps by In, As, Ga, and Sb and individual maps for each element, including some thickness measurements. EDX integrated atomic percentage spectrum profiles registered from the bottom to the top of the mapped areas shown after filtering them, in Sample A (**m**) and B (**n**).

Studies of aspects like intermixing within the superlattice or the chemical homogeneity of the bilayers must be carried out by resorting to suitable analytical techniques. We chose EDX under STEM conditions to analyze the chemical composition of large areas (i.e. above 100 nm × 100 nm) within the superlattices. Figure 4 compiles selected results regarding these EDX studies in Sample A (Fig. 4a-f) as well as in Sample B (Fig. 4g-l). The electron beam is still parallel to the same zone axes in each case as in Fig. 2. For each sample, the following information is presented from left to right. First, a representative high-angle annular dark-field (STEM-HAADF, Fig. 4a and g) image from a square-shaped area of about 60 nm × 60 nm in the middle region of the superlattice is included. In the case of Sample B, the image was taken from a region where no evident lattice defects were present to carry out a more realistic comparison between samples. Also, the regions from where these map fragments were extracted were analyzed under conditions as similar as possible (e.g. dwell time, resolution, frame registration time etc.) to further improve the reliability of the evaluation.

Secondly, Fig. 4b and h are EDX spectrum images where the signals of four different elements, which are associated with specific colors, are inserted after having confirmed their presence around these areas by EDX spectra (not shown). The intensity of these spectrum images is linked to the atomic percentage calculated by the software at each point. In other words, the more intense color is, the higher proportion of that element is estimated there. The four-element maps that form the combined spectrum images are depicted individually in Fig. 4c–f (Sample A) and 4i-l (Sample B) to identify the bilayers of the T2SL more clearly. It is worth remarking that the contrast in STEM-HAADF images is now inverted with respect to the ones shown in Fig. 2 (i.e. InAs is now the darkest layer and GaSb is the brightest). Furthermore, to improve the accuracy of the quantification result, we established a cross-section thickness of 100 nm to take into account X-Ray absorption correction and applied an optimized spectrum fit by the analysis software. Generally speaking, from the comparison of both rows of images, it is reasonable to assume that each element is predominantly present in specific regions (e.g. In and As mostly detected in the same area), thus confirming that the growth of the superlattices was successful. Some additional measurements of InAs and GaSb are included in those maps of Fig. 4 with elements whose presence should be limited, in principle, to their respective layers (i.e. As and Ga, since In and Sb are also present as InSb) to carry out a more accurate comparison. When the values obtained in Sample A are compared to those in Sample B, it is possible to observe that the InAs/GaSb thickness in the T2SL of the former looks generally more uniform than that of the latter, although the values in both layers are reasonably close to the expected ones for both T2SLs (i.e. about 5 nm, as commented previously) as shown previously in HRTEM images. However, these EDX maps help to improve the reliability of these measurements, since they are reducing the ambiguities in pinpointing the position of the main layers.

The presence of the InSb interlayers can also be predicted since overlapping signals from both indium and antimony between the main layers (i.e. InAs and GaSb) are present. Furthermore, Sample B seems to contain sharper transitions between layers than Sample A, an effect that can be observed more clearly, for instance, in the maps for indium (Fig. 4c) and i) and gallium (Fig. 4e–k). However, when the contributions of all the elements are combined (Fig. 4b and h) the opposite conclusion is reached. Namely, in Sample A, the color separation is better, whereas the signals in Sample B led to a more saturated map. Thus, it is reasonable to conclude that at least one of the mapped elements must be more widely spread throughout the superlattice than the others in Sample B. Consequently, we expect that intermixing/segregation effects across the layers must have appeared more prominently in this case than in Sample A. We can also assume that the reduced structural quality (e.g. by the presence of extended lattice defects) of Sample B must have motivated, to a certain extent, the formation of a chemically more heterogeneous superlattice. In any case, the identification of InSb with only these maps is a challenging task that requires more reliable support by another approach.

In order to complement the EDX results presented thus far, Fig. 4m and n are shown. These are spectrum profiles for Samples A and B, respectively. In each profile, each point is constituted by the integrated counts of each slice along the mapped area. This area is defined by the whole images that are shown in Fig. 4a and g. After the quantification, the variations in atomic percentage are plotted against the distance from the bottom to the top of each image. It should be pointed out that these profiles come from the maps previously shown, but after processing them with specific filtering tools available in the EDX software used. These operations were carried out to show smoother, easier to read profiles. The resulting filtered maps are not shown in this work. As a guide to the eye, some peaks that belong to either InAs or GaSb are labelled, allowing to pinpoint the position where each bilayer is situated more easily. The approximated regions with the InSb interlayers can also be indirectly found when the signals by indium and antimony are considered together; their respective profiles do not fall abruptly between InAs/GaSb peaks. Instead, some small, complementary variations on the profiles (i.e. In increases and Sb decreases or vice versa) and simultaneous slope changes can be observed in both profiles, as the ranges marked by the “InSb” labels indicate as examples. Another feature about these curves worth pointing out is that the peak distributions in the profiles from both samples match with the growth sequence selected for these T2SLs. In these EDX spectrum profiles, the sequence of InAs-InSb-GaSb-InSb-InAs is comparable to the one identified in another work on InAs/GaSb T2SLs with InSb deposited at both interfaces (i.e. GaSb-on-InAs and InAs-on-GaSb)^59^.

Furthermore, we found that profiles by indium and antimony are generally broader around their maxima and reach higher values than those from arsenic and gallium. Such a feature supports the enrichment of the T2SL in In and Sb caused by the presence of InSb. Nevertheless, the resolution of the maps available in the experiments carried out thus far barely allows us to completely discard the existence of a certain degree of atomic intermixing/segregation in either sample. However, as commented before, we expect that Sample B is the one most susceptible to these phenomena since it exhibits a lowered material quality. In this sense, the asymmetry in some peaks (e.g. indium signal in the third peak labelled as “InAs” in Fig. 4n) must be an additional hint which supports the presence of these features and also the fact that the GaSb-on-InAs and InAs-on-GaSb interfaces might not always exhibit the same width^60^, even though there is InSb at both regions. On the other hand, we can also conclude that Sample A exhibits a higher compositional homogeneity than Sample B according to these analyses. This can be verified by comparing the variations in each element curve^53,60,61^. Taking signals from arsenic as an example, it can be seen that, in Sample A, these signals drop to lower values and more abruptly than those from the same element in Sample B when the GaSb layer starts to appear on the profile. Consequently, the transitions between films must be generally sharper in Sample A, which indicates that the elements constituting its superlattices are better distributed according to the established growth process than in Sample B. For this second sample, we can assume that the future optimization of the GaSb buffer layer will contribute towards a reduction of pernicious compositional defects, and thus lead to a compositionally more homogeneous T2SL. Nevertheless, the analysis of all these composition-related issues will be explored more thoroughly in future works since it will also require a higher refinement of the EDX quantifications.

### Device characterization

**Figure 5:**
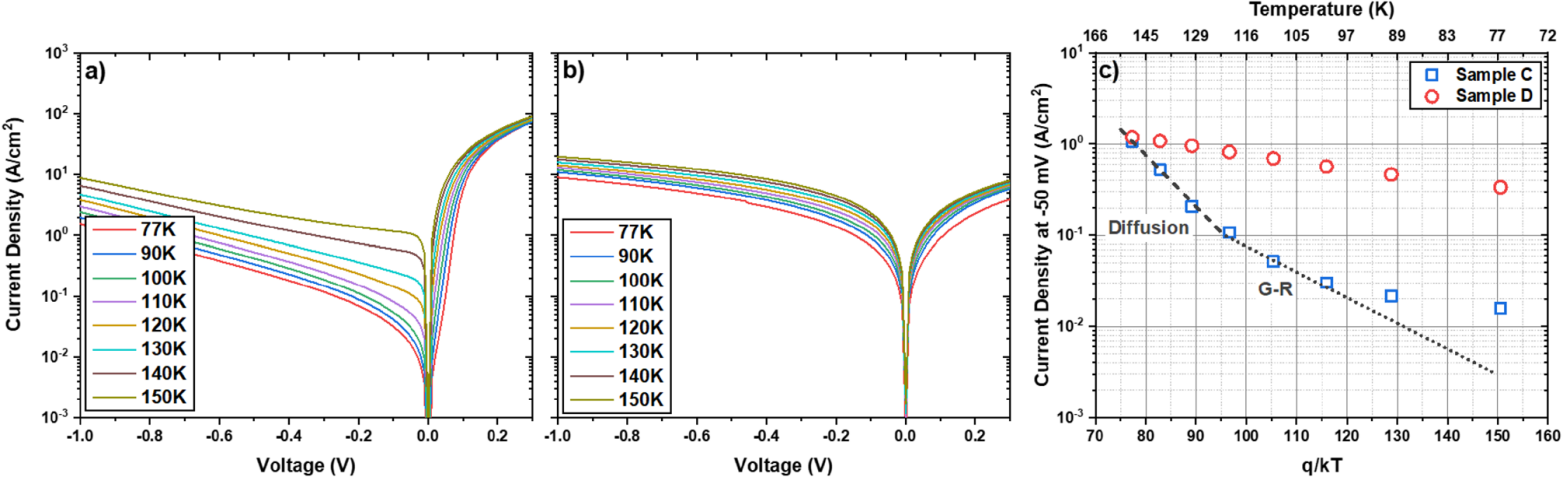
Dark current density for the temperature range 77 K–150 K for Sample C (**a**) and Sample D (**b**)﻿. Arrhenius plot of current density at −50 mV for Samples C and D (**c**).

Two p-i-n diodes were then grown on a GaSb substrate (Sample C) and a GaAs substrate using the IMF array (Sample D). In each sample, the GaSb buffer layer was followed by the growth of a 60 nm P-region, 1 μm non-intentionally doped SL absorber region, 60 nm SL N-region, and 20 nm N-doped InAs cap. All SL regions used the 12/4 SL structure and the P- and N-regions each targeted a doping concentration of 1 × 10^18^ cm^-3^. Following a standard photolithography fabrication process described in the methods section, temperature-dependent current–voltage measurements were then performed on each sample as shown in Fig. 5a and b. Figure 5c shows the Arrhenius plot of the dark current density at −50 mV for both samples. The diffusion- and generation-recombination(G-R)-limited behavior, fitted according to the analytical expressions presented by Gopal et al.^62^, are also shown. As Fig. 5c shows, Sample C is dominated by diffusion currents at temperatures above 120 K and G-R currents in the temperature range 100 K–120 K in a manner consistent with previously reported devices^47,63^. At temperatures below 100 K, the measured dark current density deviates from the fitted G-R-dominated behavior, possibly due to the presence of other sources of dark current such as trap assisted tunneling (TAT) or shunt currents. Figure 5c also shows that Sample D does not follow the expected behavior for a diffusion- or G-R-limited diode at any temperature. This suggests that either this sample does not function as a diode and we are simply measuring a path of least resistance or that the shunt current in this sample is so large as to dominate at every temperature.

**Figure 6:**
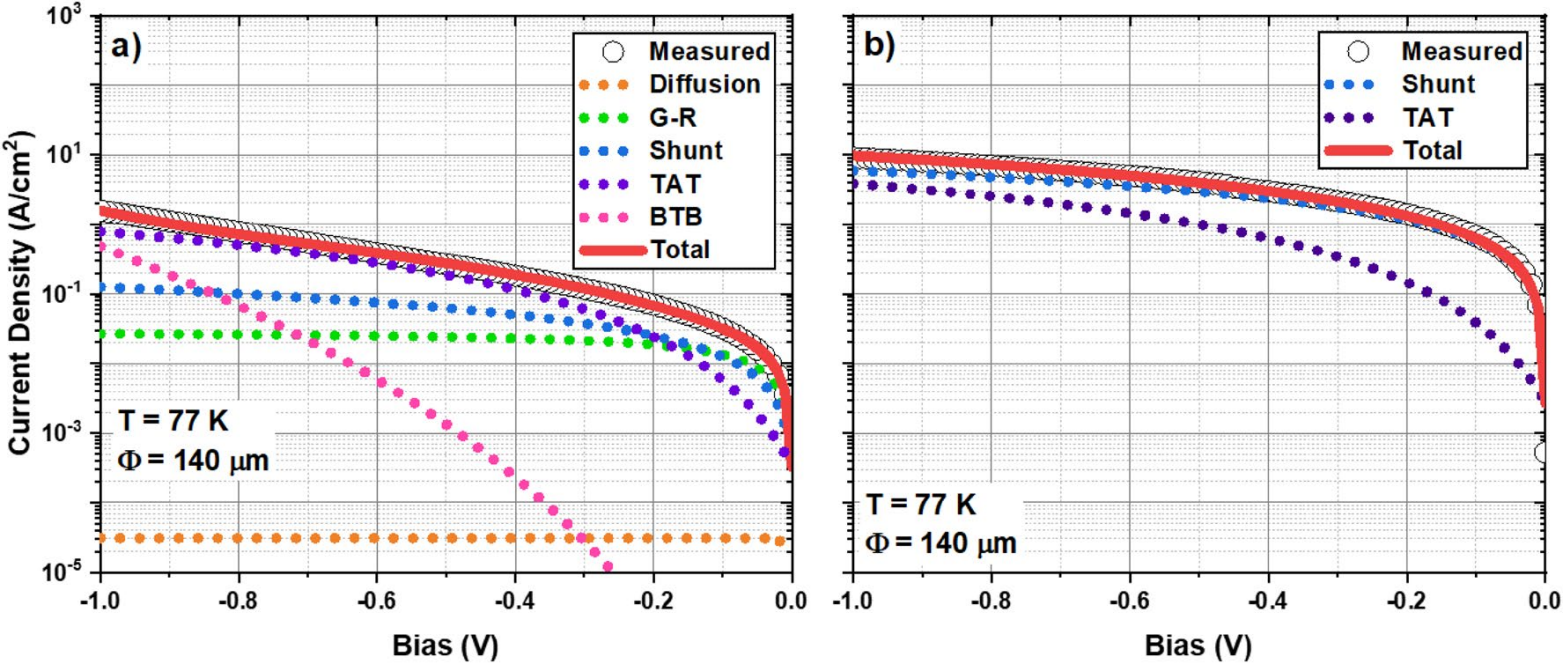
The measured and fitted dark current densities at 77 K for Sample C (**a**)and Sample D (**b**).

To confirm these hypotheses and further investigate the limiting dark current mechanisms in each diode, the analytical expressions described by Gopal were fitted to the 77 K dark current density profiles of each sample as shown in Fig. 6. Contributions from diffusion, G-R, shunt, TAT, and band-to-band tunneling (BTB) currents were considered. As shown in Fig. 6a, the reverse bias behavior of Sample C is typical of a T2SL p-i-n diode in that at low bias and low temperature the G-R current is dominant whereas TAT currents dominate as the bias increases. The shunt current provides a significant contribution which can be attributed to the lack of chemical passivation. While the problem of surface-related shunt current was here addressed through the use of a photoresist to block the mesa sidewalls from ambient air, this issue could be more effectively mitigated through chemical techniques such as dielectric deposition^64^, sulphidation^65^, or gating^66^. This explains the deviation from purely G-R-dominated behavior at low temperature observed in Fig. 5c. Furthermore, the diffusion current provides a negligible contribution at low temperatures which corroborates previous reports as well as the behavior suggested by Fig. 5c. Unlike Sample C, Sample D appears to be dominated by the shunt current at all temperatures with a minor contribution from the TAT current at high reverse bias. This seems to confirm the hypothesis that the diodes fabricated from Sample D are non-functioning. The increased prevalence of the TAT current in Sample D, as evidenced by a comparison of Fig. 6a and b, can be attributed to the increased trap density brought about by the degradation in material quality. By comparing the TAT current curves in Fig. 6a and b, we estimate that sample D has a trap density almost four times higher than that of Sample C.

**Figure 7:**
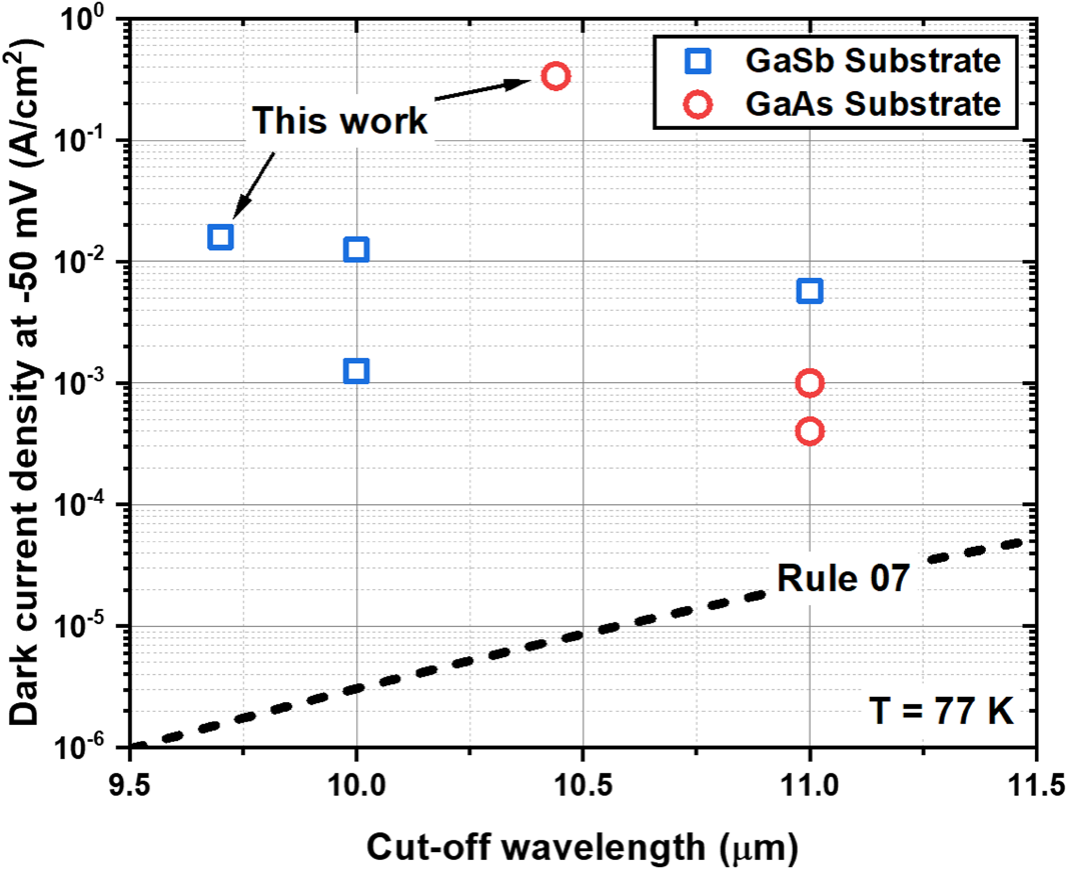
The dark current density at −50 mV of Samples C and D compared to a sampling of previously reported LWIR InAs/GaSb T2SL p-i-n diodes and the Rule 07.

The dark current density at −50 mV was measured to be 1.59 × 10^–2^ A/cm^2^ and 3.37 × 10^–1^ A/cm^2^ for Samples C and D respectively. Figure 7 compares the dark current densities at 77 K of Samples C and D with previously reported InAs/GaSb T2SL p-i-n diodes on both GaSb and GaAs substrates^13,67,68^. Rule 07^69^, a heuristic predictor for the state-of-the-art performance of an MCT diode, is also shown. Figure 7 shows that Sample C has a performance comparable to other LWIR p-i-n diodes despite the lack of chemical passivation. However, a disparity of over two orders of magnitude exists between Sample D and the p-i-n diodes on GaAs reported by Abdollahi Pour et al.^13^. However, this result was only achieved through the use of a very thick 4 μm buffer layer. It may be possible to bridge this disparity without resorting to thick buffer layers through the use of additional strain compensation techniques such as dislocation filters.

## Discussion

12/4 T2SL p-i-n diodes with cut-off wavelengths close to 10 μm have been grown on GaSb and GaAs substrates. In the latter case, the IMF array was used to prevent the propagation of dislocations into the SL region. PL and AFM measurements indicate degradation of both optical and structural quality of the T2SL on GaAs despite the presence of the IMF array. TEM measurements corroborate some of these findings since, despite the reasonably good quality of both samples in terms of crystallinity, it is observed that using a GaAs substrate led to a more defective heterostructure with an increased density of linear and planar lattice defects observable at several regions of the heterostructure: from the GaSb-on-GaAs interface to the surface of the superlattice. From the compositional point of view, the current analyses by EDX carried out allowed us to find that atomic segregation/intermixing phenomena must be mostly present in Sample B since a higher chemical homogeneity seems to have been achieved on the GaSb-based heterostructure (Sample A). We believe that this is an additional consequence derived from using a GaAs substrate together with a still not fully optimized buffering system. However, regardless of the substrate selected, the materials are deposited by the established growth sequence. Analysis of current–voltage measurements performed at cryogenic temperatures suggests that the degradation in material quality, caused by the heteroepitaxial growth of the T2SL on GaAs, undermines the diode characteristics of Sample D. It is therefore concluded that the IMF array is only partially effective as a means of mitigating the strain-induced degradation of material quality introduced by the non-native GaAs substrate. As a result, additional strain compensating measures, such as dislocation filters, are required along with the IMF array to suppress threading dislocations in the T2SL on GaAs to achieve optimum device performances.

## Methods

### Sample growth

All samples were grown by MBE in a reactor equipped with dual filament SUMO Knudsen effusion cells for Ga and In and Mark B valved cracker effusion cells for As and Sb under the same growth conditions. The InAs and GaSb layers were grown using a V/III flux ratio calibrated from RHEED oscillations of 1.2 and 2, respectively. A strain compensating InSb interlayer was grown at both interfaces of each sample using migration enhanced epitaxy with a thickness of 10% of the InAs layer.

### Photoluminescence

PL measurements were performed using a Fourier Transform Infrared Spectrometer (FTIR), equipped with a KBr beam splitter and liquid nitrogen cooled MCT-A detector. A 785 nm diode laser was used to optically pump the sample. A pattern generator was used to modulate the pump laser at a frequency of 20 kHz and a lock-in amplifier was used to subtract the unmodulated signal.

### Transmission electron microscopy

The inspection of Samples A and B by transmission electron microscopy (TEM) was carried out by observing a cross-section preparation from each heterostructure. These preparations were obtained as electron-transparent lamellae by focused ion beam (FIB) using a Zeiss Auriga FIB-SEM dual-beam electron microscope. The acceleration voltage was 30 kV, and a progressively lower ion beam current is selected for the ion-milling (from 600 to 10 pA). Afterwards, the FIB lamellae were characterized by operating a Thermo Scientific Talos F200X scanning transmission electron microscope at an acceleration voltage of 200 kV. TEM microscopy images and spectra were processed and analyzed using mainly Velox software, whereas Digital Micrograph was used to import and process electron diffraction patterns.

### Diode fabrication and characterization

A standard photolithography process was used to fabricate mesa diodes. After solvent cleaning, a bilayer photoresist was patterned onto the sample surface. Cr/Au (20:300 nm) top contacts were then thermally evaporated onto the sample. The photoresist and unwanted metals were then removed using an 1165/ acetone lift-off and an ultrasonic bath. Following patterning with a photoresist, mesas of diameters ranging from 140 μm to 440 μm were defined using a citric acid-based wet etch. The photoresist was then spun onto the mesa sidewalls, patterned, and hard-baked to protect the surface from ambient air. Finally, a Cr/Au (20:300 nm) bottom contact was thermally evaporated onto the underside of the sample. Samples were placed in a liquid nitrogen-cooled cryogenic probe station for electrical characterization.
